# Differences in Innate Cytokine Responses between European and African Children

**DOI:** 10.1371/journal.pone.0095241

**Published:** 2014-04-17

**Authors:** Lucja A. Labuda, Sanne E. de Jong, Lynn Meurs, Abena S. Amoah, Moustapha Mbow, Ulysse Ateba-Ngoa, Alwin J. van der Ham, André C. Knulst, Maria Yazdanbakhsh, Ayola A. Adegnika

**Affiliations:** 1 Department of Parasitology, Leiden University Medical Center, Leiden, The Netherlands; 2 Centre de Recherches Médicales de Lambaréné, Lambaréné, Gabon; 3 Institut für Tropenmedizin, Universität Tübingen, Tübingen, Germany; 4 Department of Biomedical Sciences, Institute of Tropical Medicine, Antwerp, Belgium; 5 Department of Parasitology, Noguchi Memorial Institute for Medical Research, University of Ghana, Legon, Accra, Ghana; 6 Immunology Department of the Laboratory of Bacteriology and Virology of Aristide Le Dantec University Hospital, Dakar, Senegal; 7 Department of Dermatology/Allergology, University Medical Center Utrecht, Utrecht, The Netherlands; University British Columbia, Canada

## Abstract

Although differences in immunological responses between populations have been found in terms of vaccine efficacy, immune responses to infections and prevalence of chronic inflammatory diseases, the mechanisms responsible for these differences are not well understood. Therefore, innate cytokine responses mediated by various classes of pattern-recognition receptors including Toll-like receptors (TLR), C-type lectin receptors (CLRs) and nucleotide-binding oligomerisation domain-like receptors (NLRs) were compared between Dutch (European), semi-urban and rural Gabonese (African) children. Whole blood was stimulated for 24 hours and the pro-inflammatory tumor necrosis factor (TNF) and the anti-inflammatory/regulatory interleukin-10 (IL-10) cytokines in culture supernatant were measured by enzyme-linked immunosorbent assay (ELISA). Gabonese children had a lower pro-inflammatory response to poly(I:C) (TLR3 ligand), but a higher pro-inflammatory response to FSL-1 (TLR2/6 ligand), Pam3 (TLR2/1 ligand) and LPS (TLR4 ligand) compared to Dutch children. Anti-inflammatory responses to Pam3 were also higher in Gabonese children. Non-TLR ligands did not induce substantial cytokine production on their own. Interaction between various TLR and non-TLR receptors was further assessed, but no differences were found between the three populations. In conclusion, using a field applicable assay, significant differences were observed in cytokine responses between European and African children to TLR ligands, but not to non-TLR ligands.

## Introduction

Geographical variations in responses to vaccines have been reported; for example, protection against pulmonary tuberculosis by the Bacillus Calmette–Guérin (BCG) vaccine varies from 0% to 80% in adults, with higher protection in populations living at higher latitudes [1], or the rotavirus vaccine which provided little protection against rotavirus diarrhea in Gambian infants but showed promising results in Europe [2]. With respect to immunological changes following vaccination, a study in infants and adolescents from the United Kingdom showed that BCG vaccination induced a marked increase in interferon-γ (IFN- γ) response to *Mycobacterium tuberculosis* purified protein derivative (Mtb PPD), but not in infants and adolescents from Malawi [3]. Differences in the innate immune system and immune responsiveness caused by factors such as genetic variability and/or environmental exposures are thought in part to underlie the varying vaccine efficacies between populations.

Pattern-recognition receptors (PRRs) enable the innate immune system to recognize pathogens through interaction with pathogen-associated molecular patterns. Activation of the innate immune system via the PRRs induces cytokine production and expression of costimulatory molecules, which in turn control the activation of the adaptive immune system [4], [5]. Several PRR families have been identified, including the Toll-like receptors (TLRs), the cytosolic nucleotide-binding oligomerisation domain (NOD)-like receptors (NLRs) and the cell-surface C-type lectin receptors (CLRs), each with their own specificities and signaling pathways. Integration of information from multiple PRRs enables the immune system to tailor its response, for example through cytokine production, to counteract specific exogenous and endogenous dangers [5], [6]. As PRRs form the starting point of the innate immune response, which ultimately shapes the adaptive immune response, the magnitude as well as the type of cytokines (*e.g.* pro- or anti-inflammatory) produced in response to stimulation of these receptors could have a major impact on health. Alterations in PRR responses can determine responses not only to vaccines or pathogens but also to commensals, allergens or self-antigens [7]–[9].

Geographical differences in PRR responsiveness might also be linked to inflammatory-related diseases that are found to differ between populations. In high-income countries, allergic diseases like asthma, hay fever and eczema [10], and chronic inflammatory disorders such as type 1 diabetes mellitus [11], [12], multiple sclerosis and inflammatory bowel disease [13] have increased, while the prevalence of these diseases is still low in low-income countries [14]–[16]. Although a better understanding of immunological differences could aid in the development of population-specific vaccines and treatment modalities against infectious, allergic and chronic inflammatory diseases, few studies have so far compared PRR responsiveness between populations.

The present study is part of the SCHISTOINIR project that aims to explore innate immune responses in schistosomiasis (www.york.ac.uk/res/schistoinir). The aim of this study was to compare PRR responsiveness between European, semi-urban African and rural African populations. To that end, pro- and anti-inflammatory responses to various TLR and non-TLR ligands were compared, using identical methods and reagents, between children living in the Netherlands, and those living in semi-urban and rural Gabon. Significant differences in TLR responsiveness in whole blood were found, with a reduced pro-inflammatory response to TLR3 stimulation and enhanced pro-inflammatory response to TLR2/1, TLR2/6 and TLR4 stimulation for Gabonese children as compared to Dutch children.

## Materials and Methods

### Ethics Statement

The study was approved in Gabon by the Comité d’Ethique Régional Indépendant de Lambaréné (CERIL N°06/08) and in the Netherlands by the UMC Utrecht Medical Research Ethics Committee. Written informed consent was obtained from parents or legal guardians of all children participating in the study.

### Study Population

In June–July 2008, 15 schoolchildren were recruited from Lambaréné, a semi-urban municipality in Gabon, and 30 children from the neighboring rural village Zilé in which *Schistosoma haematobium* infection is endemic. Lambaréné [17] and Zilé [18] have been previously described in detail.

*S. haematobium* infection was determined prior to blood collection by examining a filtrate of 10 mL of urine passed through a 12-µm-pore-size filter (Millipore, Billerica, MA, USA). Children were classified *S. haematobium*-infected when at least one *S. haematobium* egg was detected in the urine, or uninfected when three consecutive urine samples were negative. Infections with intestinal helminths *Ascaris lumbricoides* and *Trichuris trichiura* were determined by analyzing one fresh stool sample using the Kato-Katz method [19]. Hookworm larvae were determined in a 7-day coproculture of the same stool sample [20]. Infection with *P. falciparum* and microfilaria was determined by Giemsa-stained thick blood smears [21].

After collection of blood samples, all *S. haematobium*-infected children were treated with a single dose of praziquantel (40 mg/kg), and those with intestinal helminths received a single dose of albendazole (400 mg) in accordance with the guidelines of the World Health Organization. Children with fungal skin infections received therapy according to local guidelines.

Hematological parameters were analyzed using ADVIA 120 Hematology System (Bayer HealthCare LLC, Diagnostics Division, Tarrytown, NY, USA) and erythrocyte sedimentation rate was measured manually.

In September–October 2008, peripheral blood samples from 21 Dutch children, participating as controls for allergic children in a European study (EuroPrevall, www.europrevall.org), were also collected [22]. No hematological or parasitological data was available for this population, although exposure to helminths and malaria is thought to be unlikely.

### Whole Blood Culture

Heparinized venous blood was processed for culture within 6 hours after venipuncture. Whole blood was diluted 2 times in RPMI 1640 medium (Invitrogen, Carlsbad, CA, USA) supplemented with 100 U/mL penicillin (Astellas Pharma BV, Leiden, the Netherlands), 10 µg/mL streptomycin (Sigma-Aldrich, Saint Louis, MO, USA), 1 mM pyruvate (Sigma-Aldrich) and 2 mM L-glutamine (Sigma-Aldrich) and stimulated with 50 µg/mL polyinosinic-polycytidylic acid high molecular weight (poly(I:C); InvivoGen, San Diego, CA, USA), 50 ng/mL Pam2CGDPKHPKSF (FSL-1; InvivoGen), 100 ng/mL Pam3CSK4 (Pam3; EMC Microcollections GmbH, Tübingen, Germany), 100 ng/mL ultrapure lipopolysaccharide (LPS; InvivoGen), 10 µg/mL γ-D-Glu-mDAP (iE-DAP; InvivoGen), 100 µg/mL mannan (Sigma-Aldrich), 100 µg/mL curdlan (Wako Chemicals GmbH, Neuss, Germany) or 5 ng/mL 1-(palmitoyl)-2-(5-keto-6-octene-dioyl)phosphatidylcholine (KOdiA-PC; Cayman Chemicals, Ann Arbor, MI, USA), alone or in combination (Table 1). Medium was used as a negative control and 2 µg/mL phytohemagglutinin (PHA; Remel, Dartford, UK), a mitogen, as a positive control. 100 µL of ligand(s) in medium was added to wells containing 100 µL of diluted blood in 96-well round-bottom plates (Nunc; Roskilde, Denmark) and incubated in the presence of 5% CO_2_ at 37°C for 24 hours. Supernatants were harvested and stored at −80°C.

**Table 1:** Ligands used in the study, their receptors and adapters.

Ligand	PRR	TLR adapter
Poly(I:C)	TLR3	TRIF
FSL-1	TLR2/6	MyD88
Pam3	TLR2/1	MyD88
LPS	TLR4	MyD88, TRIF
iE-DAP	NLR: NOD1	-
KOdiA-PC	CD36/SR-B2	-
mannan	CLR: MR, DC-SIGN	-
curdlan	CLR: Dectin-1	-

PRR, pattern-recognition receptor. TLR, toll-like receptor. NLR, NOD-like receptor. NOD, nucleotide-binding oligomerisation domain-containing protein 1. SR, scavenger receptor. CLR, C-type lectin receptor. MR, mannose receptor. DC-SIGN, dendritic cell-specific intercellular adhesion molecule-3-grabbing non-integrin. TRIF, TIR-domain-containing adapter-inducing interferon-β. MyD88, myeloid differentiation primary response gene 88.

### Cytokine Analysis

Tumor necrosis factor (TNF) and interleukin-10 (IL-10) levels were measured in supernatants by an enzyme-linked immunosorbent assay (ELISA) according to manufacturers’ instructions using half of the reaction volume (Sanquin, Amsterdam, the Netherlands). Levels below detection limit were replaced by half the detection limit, 1.4 pg/mL and 1.2 pg/mL for TNF and IL-10 respectively.

### Statistical Analysis

Data analysis was performed using IBM SPSS Statistics version 20 for Windows (Armonk, NY, USA: IBM Corp.; 2011). Cytokine concentrations in response to stimulation were corrected for spontaneous cytokine production by subtracting medium responses. Negative values and zeros were subsequently replaced by the lowest positive value, 0.08 and 0.04 for TNF and IL-10 respectively. The pro/anti-inflammatory TNF/IL-10 ratio was calculated by: (TNF^stimulated^ − TNF^medium^)/(IL-10^stimulated^ − IL-10^medium^). The degree of interaction between two ligands was calculated for *e.g.* poly(I:C) and iE-DAP by: (TNF^poly(I:C)+iE-DAP^ − TNF^medium^)/((TNF^poly(I:C)^ − TNF^medium^) + (TNF^iE-DAP^ − TNF^medium^)). This could result in a synergistic or inhibitory effect. Mann-Whitney *U* test and Kruskal-Wallis test with Dunn’s post-test were used to compare groups. Fisher’s exact test and Pearson chi-square test were used to compare categorical data of the population characteristics. Wilcoxon signed rank test was used to compare the (synergistic) response to combined stimulation with the sum of cytokines produced in two separate stimulations (after background subtraction) and for comparing the response to stimulation with medium condition. *p*-values less than 0.05 were considered statistically significant.

Graphs were made with GraphPad Prism version 6 for Windows (La Jolla, CA, USA: GraphPad Software; 2013). Box plots have 10–90% range whiskers and bar graphs show medians.

## Results

### Study Population

The study population consisted of 21 Dutch children (median age: 9y, age range: 8–11, sex ratio: 12m/9f), 15 semi-urban Gabonese children (median age: 10y, age range: 6–15, sex ratio: 7m/8f) and 30 rural Gabonese children (median age: 10y, age range: 7–16, sex ratio: 17m/13f). The groups did not differ significantly in distributions of age and sex. Gabonese children differed in positivity for *S. haematobium*; semi-urban children were negative for schistosomiasis, whereas 57% of rural children were positive for this infection. Furthermore, 17% of semi-urban children were infected with geohelminths (*Trichuris trichiura*), while 44% of rural children were geohelminth positive (hookworm, *Ascaris lumbricoides* and/or *Trichuris trichiura*). A single case of malaria and of microfilaria and 5 cases of fungal skin infections were also detected amongst the rural Gabonese children, and 1 case of fungal skin infection amongst the semi-urban children. Rural children had higher levels of leukocyte (median: 6.50·10^9^/L vs. 9.15·10^9^/L, *p* = 0.003) and eosinophil (median: 0.28·10^9^/L vs. 1.31·10^9^/L, *p* = 0.001) counts compared to semi-urban children. While there was no parasitological data for Dutch children, exposure to helminths and malaria is unlikely. The Dutch hematological reference values are 4.5–13.5·10^9^/L for leukocyte count and <0.4·10^9^/L for eosinophil count [23].

### Cytokine Responses to TLR Ligands in European and African Children

**Figure 1:**
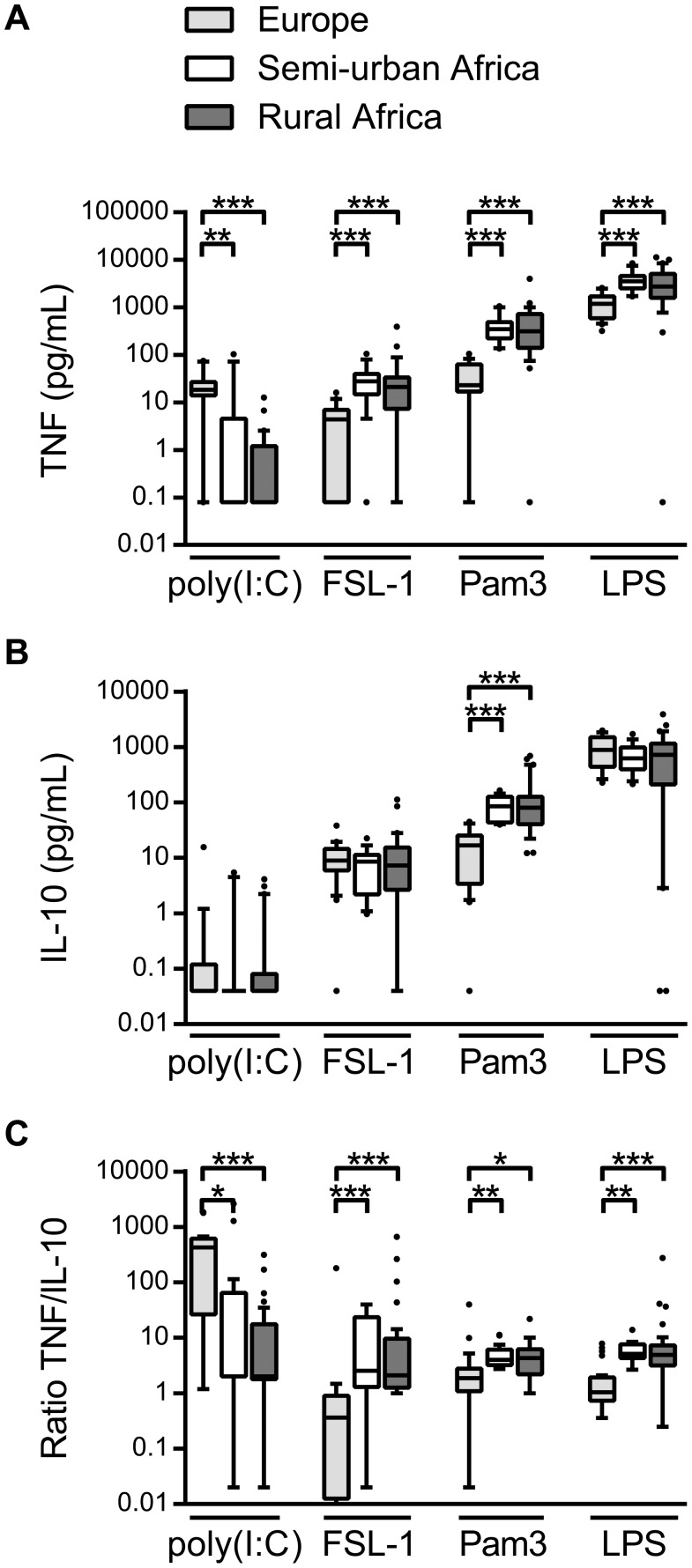
Whole blood cytokine responses to TLR ligands. A) TNF responses to poly(I:C), FLS-1, Pam3 and LPS in European children (the Netherlands), and semi-urban and rural African children (Gabon). B) IL-10 responses to TLR stimulation. C) Pro/anti-inflammatory ratio as calculated by TNF/IL-10 ratio. *p<0.05, **p<0.01, ***p<0.001.

To compare innate immune responses of Dutch children with semi-urban and rural Gabonese children, whole blood was stimulated with different TLR and non-TLR ligands. Following poly(I:C) (TLR3 ligand) stimulation, TNF responses were lower in Gabonese children as compared to Dutch children, while IL-10 responses were low for all groups (Figure 1A and B). This resulted in a lower TNF/IL-10 ratio for Gabonese children (Figure 1C). Conversely, TNF responses to FSL-1 (TLR2/6 ligand), Pam3 (TLR2/1 ligand) and LPS (TLR4 ligand) were higher in Gabonese children (Figure 1A); IL-10 responses to FSL-1 and LPS did not differ between the groups and response to Pam3 was higher in Gabonese children (Figure 1B). When considered as ratio, Gabonese children exhibited a higher TNF/IL-10 ratio in response to these TLR ligands (Figure 1C). No differences were found between semi-urban and rural Gabonese children in whole blood responses to any of the TLR ligands tested. Comparison of responses by *Schistosoma* or geohelminth infection status did not result in significant differences (data not shown).

Spontaneous production of cytokines (response to medium) was low (Supplementary figure 1A). Despite the low levels, spontaneous IL-10 production was significantly higher for rural Gabonese children as compared to Dutch children, with a similar trend for higher spontaneous TNF production. No differences were observed between the groups in response to the positive control, the mitogen PHA (Supplementary figure 1B).

### Interaction between TLR and Non-TLR Ligands in European Children

Non-TLR ligands iE-DAP (a NOD1 ligand), mannan (ligand of mannose receptor (MR) and/or dendritic cell-specific intercellular adhesion molecule-3-grabbing non-integrin (DC-SIGN)), curdlan (a Dectin-1 ligand) or KOdiA-PC (ligand of the scavenger receptor CD36/SR-B2 [24]) alone did not induce substantial TNF or IL-10 production (Supplementary figure 2). However, they did influence TLR-mediated responses significantly. A synergistic effect was observed for the production of TNF, in particular in the interaction between TLR receptors and Dectin-1, while for IL-10 production both synergistic and inhibitory effects were observed (Table 2 and Supplementary figure 3).

**Table 2:** Degree of interaction between TLR and non-TLR ligands in Dutch children.

	TNF				IL-10			
	poly(I:C)	FSL-1	Pam3	LPS	poly(I:C)	FSL-1	Pam3	LPS
iE-DAP	++		+	+		+	+	+
mannan	+	+	+				+	+
KOdiA-PC					−			+
curdlan		++	++	++		−		−

The degree of interaction between two ligands was calculated for *e.g.* poly(I:C) and iE-DAP by: (TNF^poly(I:C)+iE-DAP^ − TNF^medium^)/((TNF^poly(I:C)^ − TNF^medium^) + (TNF^iE-DAP^ − TNF^medium^)). Values above 1 are regarded as synergy (+), and above 2 as strong synergy (++). Values below 1 are regarded as inhibition (−). Shown are statistically significant differences (*p*<0.05) according to the Wilcoxon signed rank test, when comparing the response to combined stimulation with the sum of separate stimulation (after background subtraction). See Supplementary figure 3 for details.

### Comparison of PRR Interaction Profiles in European and African Children

**Figure 2:**
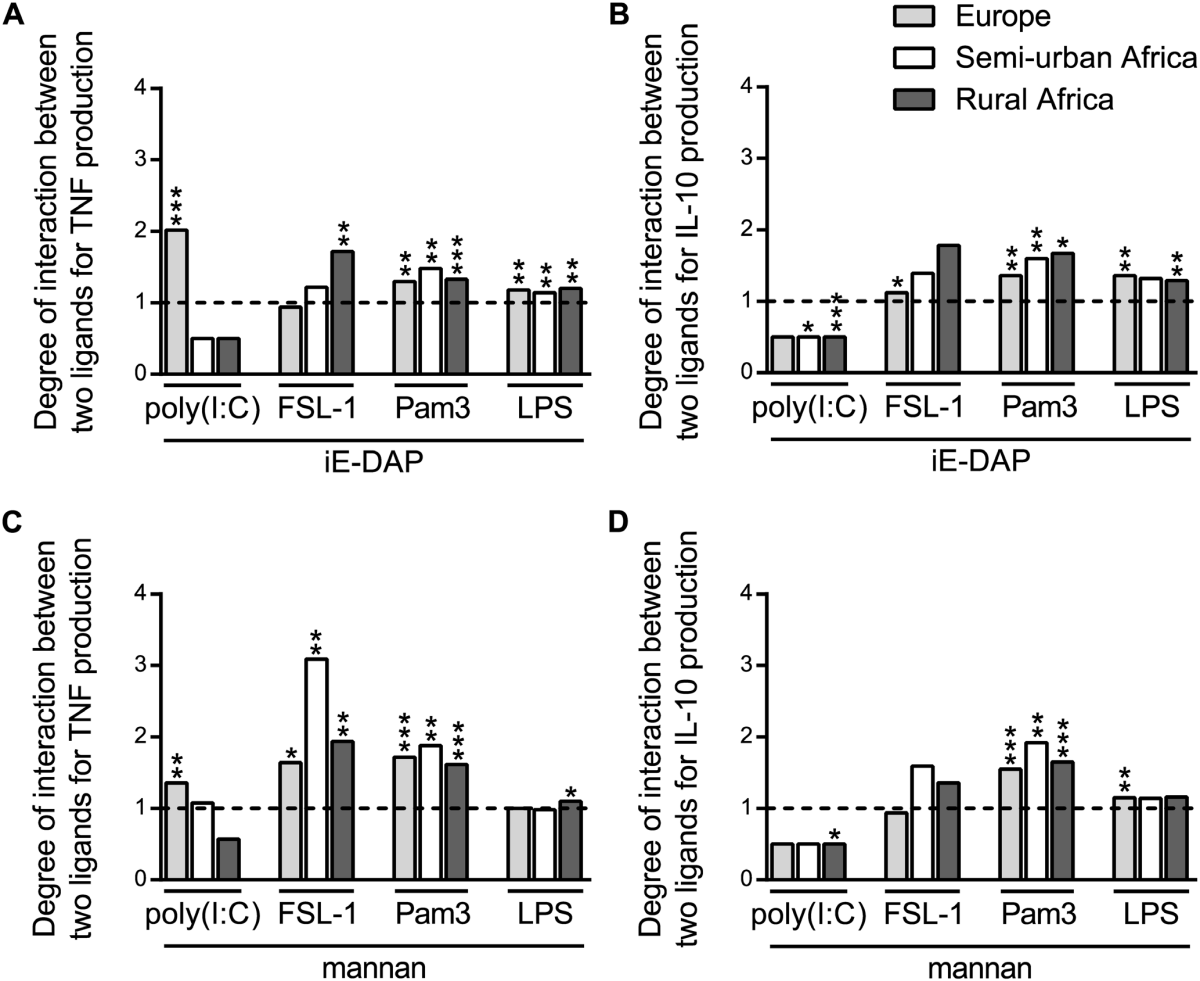
Degree of interaction between TLR and non-TLR ligands. A) Degree of interaction for TNF production upon stimulation with poly(I:C), FSL-1, Pam3 or LPS combined with iE-DAP. This was calculated for e.g. poly(I:C) and iE-DAP by: (TNF^poly(I:C)+iE-DAP^ − TNF^medium^)***/***((TNF^poly(I:C)^ − TNF^medium^) + (TNF^iE-DAP^ − TNF^medium^)). B) Degree of interaction for IL-10 production for combinations with iE-DAP. C) Degree of interaction for TNF production for combinations with mannan. D) Degree of interaction for IL-10 production for combinations with mannan.

Interactions between TLRs and NOD1 (by iE-DAP) and MR/DC-SIGN (by mannan) were further compared between Dutch children and semi-urban and rural Gabonese children. Combination of Pam3 and iE-DAP or mannan resulted in significant synergistic effects for both TNF and IL-10 in all groups of children; other combinations were more variable (Figure 2, Supplementary figure 4). Of interest, while in Dutch children combined stimulation with poly(I:C) and iE-DAP or mannan led to significant synergy, Gabonese children exhibited a tendency towards inhibitory responses.

## Discussion

This is the first study to systematically examine innate immune responses to several classes of PPRs in European and African children that originate from a semi-urban and a close-by rural area. African children from both a semi-urban and a rural area were included to take into account differences in environmental factors and genetic diversity between the African and European populations.

We report that Gabonese children had a reduced pro-inflammatory response to TLR3-ligand poly(I:C) and an enhanced pro-inflammatory response to TLR2/6-ligand FSL-1, TLR2/1-ligand Pam3 and TLR4-ligand LPS as compared to Dutch children. We further show that the concomitant engagement of TLRs with the receptors NOD1, CD36, MR/DC-SIGN or Dectin-1 can modify TLR responses, but no clear differences in the responses between Dutch and semi-urban and rural Gabonese children were seen, indicating that innate immune responses to the non-TLR ligands studied are similar in European and Gabonese children.

Geographical differences in immune responses have not been studied extensively. One of the most field-applicable methods is the whole blood assay, which has been used here and in a few other studies to profile immune responses in populations residing in different geographic areas. In a recent study comparing responses of 2-year-old children to various PRRs across four continents, Smolen *et al.* [25], have shown that despite very distinct genetic and environmental background, the cytokine responses were largely similar in European, North American and South American children. However, the response of children from Africa was poor compared with the other three continents when stimulated with ligands to surface (TLR2, TLR4) or endosomal (TLR3, TLR7/8) PRRs but similar when targeting the cytosolic PRR (NOD2). However, our results are different. First, we find higher innate responses to LPS, Pam3 and FSL-1, stimuli which engage surface TLRs, in African (Gabonese) compared to European (Dutch) children. Currently, we are unable to explain the difference between our study and that of Smolen *et al.*, but it has to be emphasized that the age of the study subjects was substantially different in the two studies and differences in immune responses between Africans and Europeans has been found to be age-dependent [26]. Second, we see differences in immune responses between the genetically-distinct European and African children, but not in the genetically-similar semi-urban and rural African children. These results would suggest that genetic differences play an important role in the distinct innate responsiveness of African and European children. Polymorphisms have been described for TLR receptors and for molecules in downstream signaling pathways, for example for the gene coding for TIR domain-containing adapter protein/MyD88 adapter-like (TIRAP/Mal) that interacts with MyD88, and in TRIF [27]. Alternatively, it is possible that the difference between the semi-urban and rural African areas studied is too small to have a substantial effect on innate immune responsiveness.

The differences we observed in cytokine production in response to TLR ligands between Dutch and Gabonese children might be explained by variations in TLR expression levels. Van den Biggelaar *et al.* analyzed cord blood mononuclear cells (CBMCs) of newborns from a low-income country (Papua New Guinea; PNG) and a high-income country (Australia), showing less TNF and IL-10 in response to TLR2- and TLR4-specific stimulation, but more IFN-γ and IL-10 in response to BCG (recognized by TLR2, TLR4, and putatively TLR9) in PNG newborns [28]. The same study measured TLR mRNA expression and found that PNG CMBCs had higher TLR2 and TLR9 expression levels, but lower levels of TLR4. In contrast, Gabonese CBMCs have been shown to have lower TLR2 expression compared to Austrian CBMCs, but this was measured on freshly isolated cells that were fixed and then analyzed by flow cytometry [29] whereas the PNG study assessed mRNA expression in cryopreserved cells. Altogether, these data indicate how important technical differences can be when comparing studies conducted by different groups.

Downstream signaling pathways may also contribute towards differences observed in TLR responsiveness. TLR3 signals via Toll-IL-1 receptor (TIR) domain-containing adaptor inducing interferon-β (TRIF), while TLR2/6 and TLR2/1 signal via myeloid differentiation primary response protein 88 (MyD88) and TLR4 signals via both [30]. Thus, the enhanced pro-inflammatory response upon TLR3 stimulation in Dutch as compared to Gabonese children may be due to altered signaling via TRIF. Similarly, the enhanced pro-inflammatory response upon TLR2/6, TLR2/1 and TLR4 stimulation in Gabonese children may result from differences in MyD88 signaling. With the emergence of evidence for ‘trained innate immunity’ where a ‘memory’ of an earlier innate triggering in natural killer cells, macrophages and monocytes can be imprinted and can affect response to an innate ligand encountered later [31], it is possible that differential exposure to viruses, bacteria and parasites in the Dutch and Gabonese populations may explain the observed differences in innate responses [32]–[34].

Interaction of TLRs with other PRRs can modulate the type and the strength of an immune response. In Dutch children, combinations with curdlan resulted in the highest degree of synergy in TNF production, as described before [35]. Interestingly, despite the strong synergy between curdlan and TLRs that engage MyD88, no synergy was seen with the TLR3-ligand poly(I:C) which engages TIR. NOD1 has been found previously to synergize with TLR3 and TLR4 to induce IL-12p70 production [36], and our data indicate that synergy (by iE-DAP) can also occur for TNF with TLR2/1, TLR3 and TLR4. Mannan was shown to synergize with TLR3, TLR2/6 and TLR2/1 for TNF production and with TLR2/6 and TLR4 for IL-10 production, in addition to the already described MR synergy with TLR2 [37]. CD36, stimulated by KOdiA-PC, did not synergize with TLRs for TNF production, while IL-10 production was variably affected.

The degree of interaction between PRRs did not differ considerably between Dutch children and those from semi-urban and rural Gabon. However, synergy between iE-DAP or mannan with poly(I:C) for TNF production occurred for Dutch children, but not for Gabonese children. This might suggest that NOD1 and MR are expressed to a lower extent in Gabonese children. However, when stimulating with LPS or FSL-1, children from rural Gabon showed higher activity of MR and NOD1 respectively. Therefore, it is unlikely that receptor expression explains the differences observed but rather the downstream pathways involved might be differently shaped.

Innate responses did not differ significantly between semi-urban and rural Gabonese children, and furthermore *S. haematobium* infection did not lead to differences in PRR responsiveness. This is in contrast to studies where helminth infections were found to influence TLR responses or expression levels [18], [34], [38], [39]. This may be due to the difference between whole blood responses measured here and isolated peripheral blood mononuclear cells used in the other studies. Indeed, a larger follow-up study carried out in the same area measuring cytokine responses in whole blood cultures did not show differences in innate responses (submitted). While whole blood assays are ideal for field conditions as cell separation is not required, their main disadvantage is that the number of cells cultured is unknown and not controlled for. In this study, leukocyte and eosinophil counts did differ significantly between semi-urban and rural Gabonese children, most likely due to the higher rate of *S. haematobium* infection in the rural area. This however did not result in significant differences in innate cytokine responses. Future studies using isolated peripheral blood mononuclear cells and intracellular cytokine staining combined with receptor expression measurements would give more detailed information about differences in immunological responses at the population level.

Our study is one of the few to compare, using identical protocols, not only children in Europe and Africa but also children from rural and semi-urban Africa. The reduced pro-inflammatory response to TLR3 stimulation and enhanced pro-inflammatory response to TLR2/1, TLR2/6 and TLR4 stimulation in African children could affect the efficacy of vaccines or treatments and should be taken into account in future studies. Furthermore, the distinct PRR responses might be of interest when trying to understand differences between populations in terms of allergic and chronic inflammatory diseases.

## Supporting Information

Figure S1 **Whole blood cytokine production in negative and positive control samples.** A) Spontaneous TNF and IL-10 production in negative control samples (medium). B) TNF and IL-10 production in positive control samples (PHA).
(TIF)

Figure S2 **Whole blood cytokine responses to non-TLR stimulation.** A) TNF production in response to non-TLR ligands (iE-DAP, mannan, KOdiA-PC and curdlan). B) IL-10 response to non-TLR ligands. *p-value when comparing two areas. ^#^p-value when comparing stimulated condition with medium condition.
(TIF)

Figure S3 **Interaction between TLR and non-TLR ligands in Dutch children.** A) TNF responses to stimulation with poly(I:C), FSL-1, Pam3 or LPS combined with iE-DAP, mannan, KOdiA-PC or curdlan. B) IL-10 responses to combined stimulations. C) Degree of interaction between two ligands for TNF production. D) Degree of interaction between two ligands for IL-10 production.
(TIF)

Figure S4 **Interaction between TLR and non-TLR ligands.** A) TNF responses to stimulation with poly(I:C), FSL-1, Pam3 or LPS combined with iE-DAP. B) IL-10 responses to TLR ligands combined with iE-DAP. C) TNF responses to TLR ligands combined with mannan. D) IL-10 responses to TLR ligands combined with mannan.
(TIF)
