# Supplementary figures and images for: Differences in Innate Cytokine Responses between European and African Children
Source: PLoS One. 2014 Apr 17;9(4):e95241. doi: 10.1371/journal.pone.0095241 (PMC3990610; doi:10.1371/journal.pone.0095241)

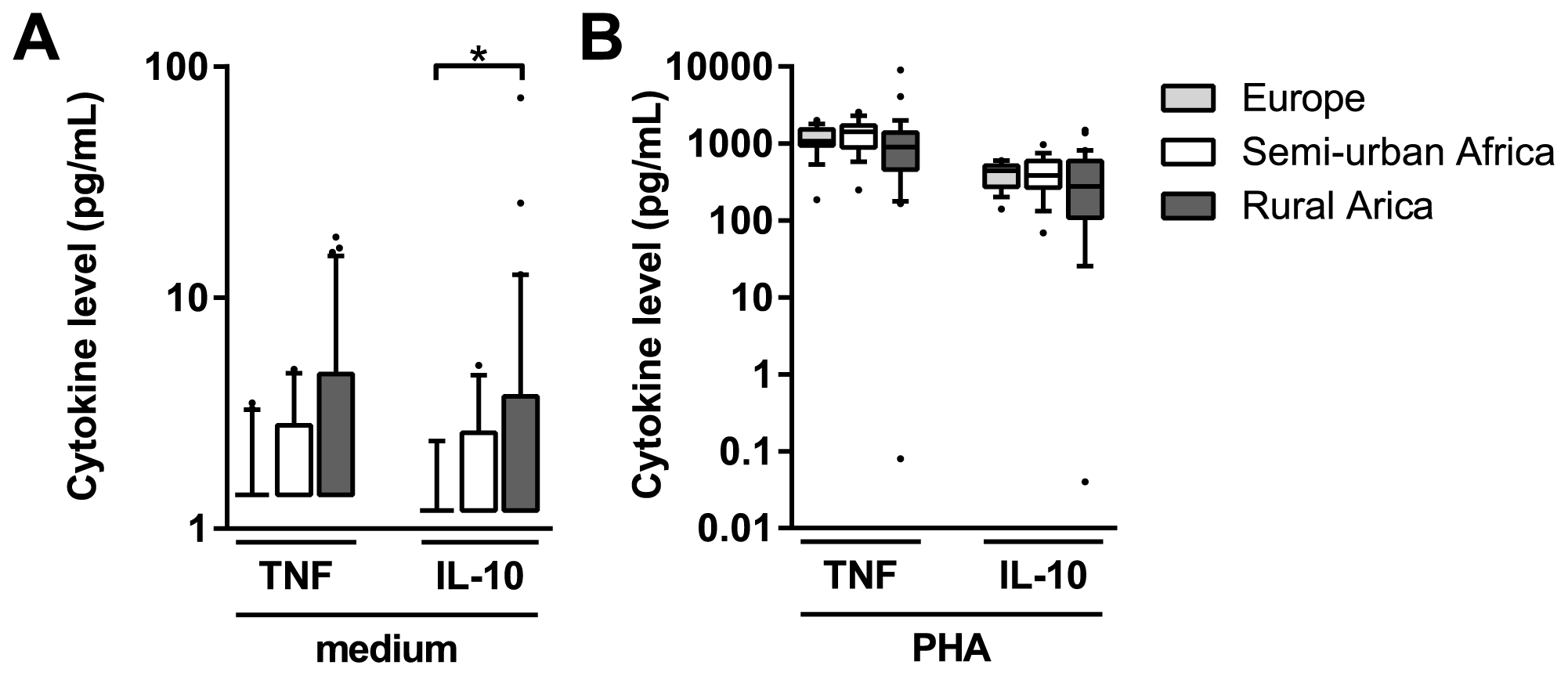

Supplement: Figure S1 — Whole blood cytokine production in negative and positive control samples. A) Spontaneous TNF and IL-10 production in negative control samples (medium). B) TNF and IL-10 production in positive control samples (PHA). (TIF) [file pone.0095241.s001.tif]

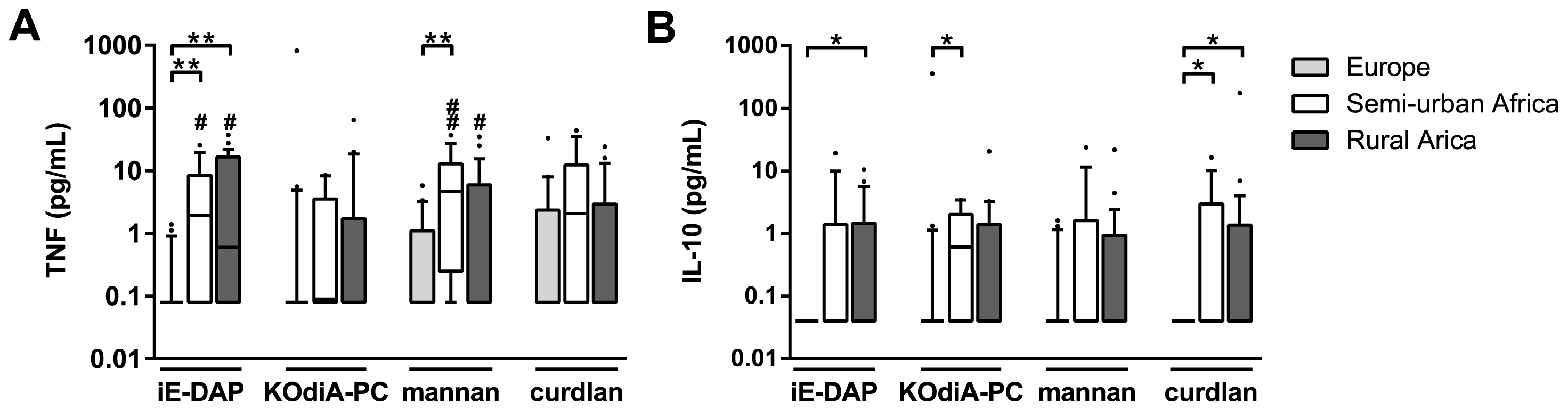

Supplement: Figure S2 — Whole blood cytokine responses to non-TLR stimulation. A) TNF production in response to non-TLR ligands (iE-DAP, mannan, KOdiA-PC and curdlan). B) IL-10 response to non-TLR ligands. *p-value when comparing two areas. #p-value when comparing stimulated condition with medium condition. (TIF) [file pone.0095241.s002.tif]

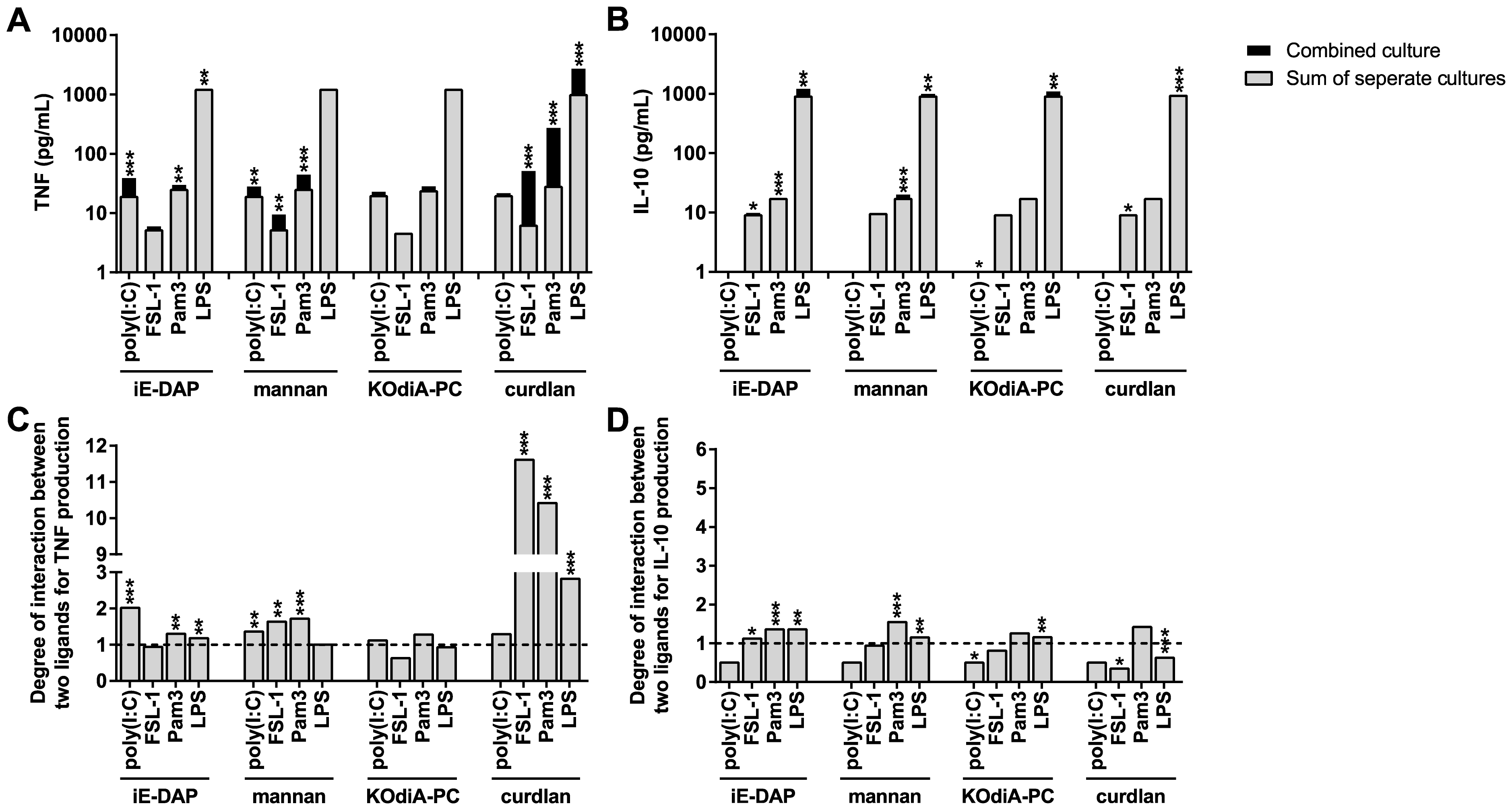

Supplement: Figure S3 — Interaction between TLR and non-TLR ligands in Dutch children. A) TNF responses to stimulation with poly(I:C), FSL-1, Pam3 or LPS combined with iE-DAP, mannan, KOdiA-PC or curdlan. B) IL-10 responses to combined stimulations. C) Degree of interaction between two ligands for TNF production. D) Degree of interaction between two ligands for IL-10 production. (TIF) [file pone.0095241.s003.tif]

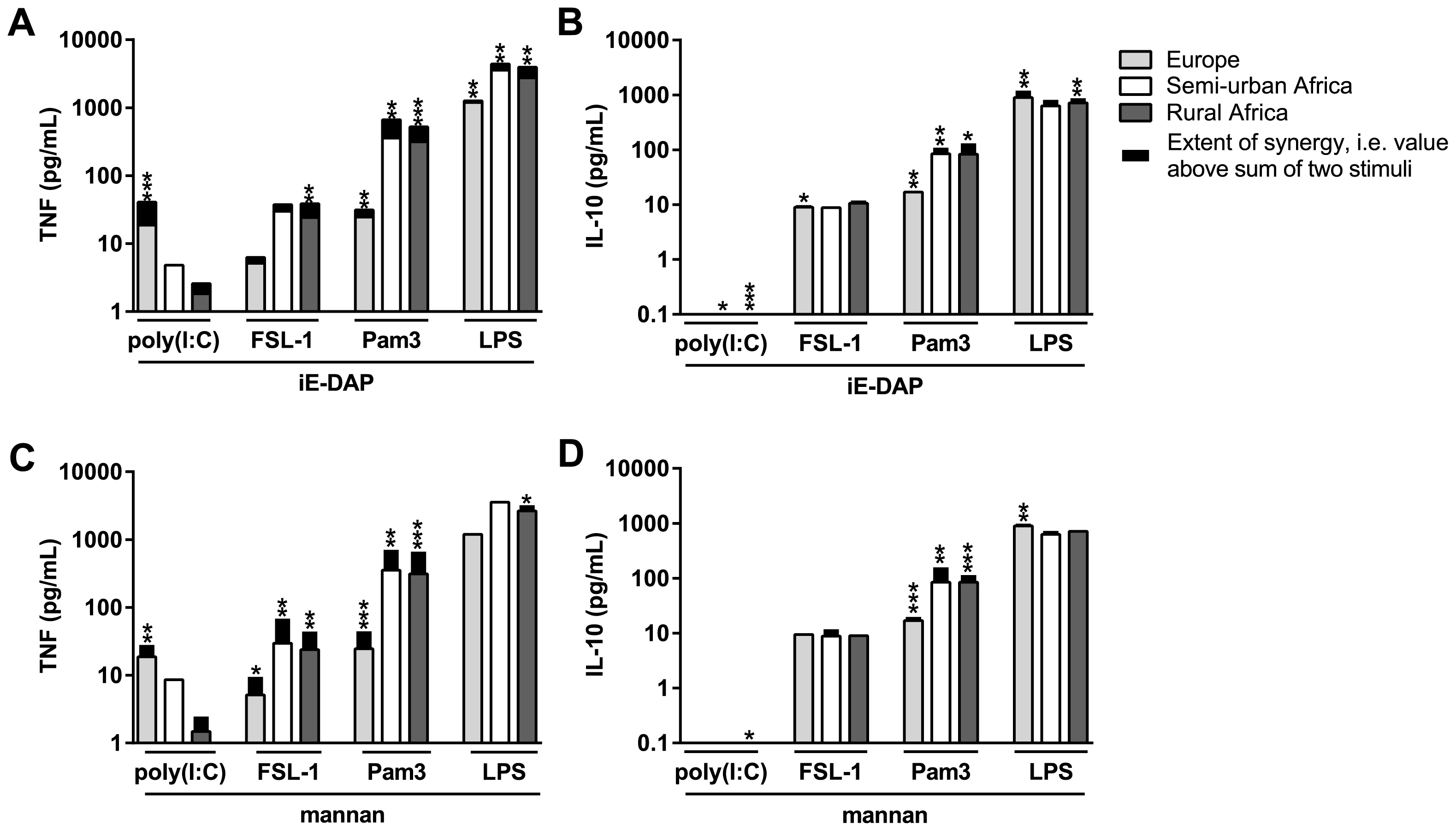

Supplement: Figure S4 — Interaction between TLR and non-TLR ligands. A) TNF responses to stimulation with poly(I:C), FSL-1, Pam3 or LPS combined with iE-DAP. B) IL-10 responses to TLR ligands combined with iE-DAP. C) TNF responses to TLR ligands combined with mannan. D) IL-10 responses to TLR ligands combined with mannan. (TIF) [file pone.0095241.s004.tif]
